# *Rhodiola rosea* Impairs Acquisition and Expression of Conditioned Place Preference Induced by Cocaine

**DOI:** 10.1155/2013/697632

**Published:** 2013-09-23

**Authors:** Federica Titomanlio, Carmen Manzanedo, Marta Rodríguez-Arias, Laura Mattioli, Marina Perfumi, José Miñarro, María A. Aguilar

**Affiliations:** ^1^Pharmacognosy Unit, School of Pharmacy, University of Camerino, Via Madonna delle Carceri 9, 62032 Camerino, Italy; ^2^Unit of Research on Psychobiology of Drug Dependence, Department of Psychobiology, School of Psychology, University of Valencia, Avenida Blasco Ibañez 21, 46010 Valencia, Spain

## Abstract

A novel approach to the treatment of adverse effects of drugs of abuse is one which makes use of natural products. The present study investigated the effect of *Rhodiola rosea* L. hydroalcoholic extract (RHO) on cocaine-induced hyperactivity and conditioned place preference (CPP) in mice. In a first experiment, mice received RHO (15, 20 or 25 mg/kg, IG), cocaine (25 mg/kg, i.p.) (COC), or a combination of both drugs (COC + RHO15, COC + RHO20, and COC + RHO25), and their locomotor activity was evaluated. In a second experiment, the effects of RHO on the acquisition, expression, and reinstatement of cocaine CPP (induced by drug priming or social defeat stress) were evaluated. RHO alone did not increase activity but potentiated the hyperactivity induced by cocaine. *Rhodiola* did not induce motivational effects by itself but attenuated the acquisition and expression of cocaine-induced CPP. Moreover, it was found that RHO did not block reinstatement. The results indicate that RHO is effective in reducing the rewarding properties of cocaine but is ineffective in preventing priming or stress-induced cocaine reinstatement. In light of these findings, the benefits of *Rhodiola rosea* L. as a treatment of cocaine addiction would seem to be limited.

## 1. Introduction

Cocaine addiction has become one of the most serious economic and health problems of developed societies, as it affects a great number of individuals [1]. Development of effective treatments for cocaine dependence is necessary to reduce its impact upon both individual and society. However, currently, there are no approved medications for cocaine dependence, despite numerous studies of pharmacologic agents in both animal and human models [2, 3]. A novel approach to the treatment of adverse effects of drugs of abuse is one which makes use of natural products. Recently, there has been an increase in the number of preclinical models and clinical trials addressing the effectiveness of such agents and their active constituents [4]. *Rhodiola rosea* L. (fam. Crassulaceae) is a well-known traditional oriental medicine with adaptogenic, anxiolytic, antidepressive, and antistress properties [5–10]. The positive effects of a *Rhodiola rosea* L. extract (RHO) in preventing nicotine and morphine withdrawal symptoms and countering the development of dependence on these drugs have recently been demonstrated in animal models [11, 12]. A recent study demonstrated that the acquisition, expression, and reinstatement of morphine-induced conditioned place preference (CPP) are blocked by RHO [13]. These results suggest that RHO is capable of reducing craving and vulnerability to relapse and might be an effective natural remedy for the treatment of opioid addiction [13]. 

The CPP paradigm has been used to evaluate the rewarding action of drugs of abuse, including cocaine [14–16], and it can be used as an animal model of relapse to drug-seeking behaviour when, after extinction of CPP, exposure to drug priming or stress induces the reinstatement of CPP [17]. In previous studies, we have demonstrated that cocaine induces CPP in mice at doses between 1 and 50 mg/kg [18–22], and that cocaine priming produces reinstatement of cocaine-induced CPP [18, 19, 21, 22]. Several stressful events also induce reinstatement of cocaine CPP [17, 23–25], including social defeat stress [26].

The present study aimed to evaluate the effects of RHO on the motor and rewarding effects of cocaine in the CPP paradigm because RHO appears to modulate the levels and activities of biogenic monoamines, such as serotonin (5-HT), dopamine (DA), and noradrenaline (NA), in the neural pathways involved in the regulation of addiction [6, 27–29]. We hypothesised that the effects of cocaine can be altered by RHO administration but in a different way to those observed with morphine, since behavioural and neurobiological differences between opiates and psychostimulants have been reported [30]. Therefore, the objective of the present work was to test the effectiveness of RHO as a treatment for cocaine addiction in an animal model. We evaluated whether treatment with RHO altered the hyperactivity and rewarding effects of cocaine (acquisition, expression, and priming- or stress-induced reinstatement of CPP) in mice. 

## 2. Materials and Methods

*Subjects*. OF1 male mice (Charles River, Barcelona, Spain) arrived at the laboratory at 42 days of age and were housed in groups of four in plastic cages (28 cm × 28 cm × 14.5 cm) for 10 days prior to the initiation of experiments, under the following conditions: constant temperature (21 ± 2°C), a light schedule (white lights on: 07.30–19.30 h), and food and water available ad libitum. Each animal was handled briefly on the 3 days preceding the initiation of experiments and acclimatised to intragastric administration. Procedures involving mice and their care were conducted in compliance with national, regional, and local laws and regulations, which are in accordance with the European Communities Council Directives of 24 November 1986 (86/609/EEC). 

*Apparatus*. Locomotor activity was automatically measured by an actimeter (CIBERTEC S.A., Spain) consisting of eight cages (33 × 15 × 13 cm), each with eight infrared lights located in a frame around the cage (body level of mice). The different frames are separated from each other by a distance of 4 cm and, since they are opaque, prevent animals from seeing cospecifics. The conditioning place preference apparatus consists of four identical Plexiglas place-conditioning boxes. Each box consists of two equally sized compartments (30.7 cm × 31.5 cm × 34.5 cm) separated by a gray central area (13.8 cm × 31.5 cm × 34.5 cm). The compartments have different coloured walls (black versus white) and distinct floor textures (smooth in the black compartment and rough in the white). Four infrared light beams in each compartment of the box and six in the central area allow the recording of the position of the animal and its crossings from one compartment to the other. The equipment was controlled by an IBM PC computer using MONPRE 2Z software (CIBERTEC, SA, Spain).

*Drugs*. A dried hydroalcoholic extract from the roots of *Rhodiola rosea* L. (RHO) was employed for the experiments (EPO S.r.l., Milan, Italy; lot number 601252). The HPLC analysis showed a content of 3% total rosavins, expressed as rosavin, 1% salidroside, and 0.8% tyrosol (for more details see [13]). The extract was dissolved in 1% v/v ethanol solution and administered by gavage (IG) at doses of 15, 20, and 25 mg/kg/10 mL. The same vehicle (1% v/v ethanol solution) was administered IG to the control groups. The doses of RHO were selected based on previous studies in which 10, 15, and 20 mg/kg blocked the effects of nicotine and morphine [11–13]. In the present experiments given that a different drug of abuse was being evaluated, we employed a higher dose of RHO (25 mg/kg) and did not employ 10 mg/kg (the less effective dose in previous studies [11–13]). Cocaine chlorhydrate (Laboratorios Alcaliber, Madrid, Spain) was dissolved in saline (NaCl 0.9%) in a volume of 0.01 mL/g and was administered intraperitoneally (i.p.) at a dose of 25 mg/kg. This dose was selected based on a previous study in which it consistently induced CPP [18]. Half of this dose was used for cocaine priming (12.5 mg/kg), also due to results obtained in the aforementioned study [18].

**Experiment 1**

Effects of RHO on cocaine-induced hyperactivity.

### 2.1. Procedure and Experimental Design

In this experiment, eight groups of mice were used (*n* = 8 per group). Spontaneous motor activity was recorded for 30 minutes (habituation, 0–30 min). Afterwards, two groups received a vehicle, two groups received 15 mg/kg of RHO, two groups received 20 mg/kg of RHO, and the last two groups received 25 mg/kg of RHO, and their activity was recorded for 60 minutes in blocks of 30 min (31–60, 61–90 min). Next, each of the two previously named groups was given either saline (groups Veh-Sal, RHO15, RHO20, RHO25) or 25 mg/kg of cocaine (Veh-Coc, RHO15-Coc, RHO20-Coc, and RHO25-Coc), and their activity was registered for a further 60 minutes (91–120, 121–150 min). 

### 2.2. Statistical Analysis

Motor activity data registered during the total 150 min register were analyzed using a mixed analysis of variance (ANOVA) with one “between-subjects” variable “treatment” with eight levels (Veh-Sal, RHO15, RHO20, RHO25, Veh-Coc, RHO15 + Coc, RHO20 + Coc, and RHO25 + Coc) and a “within-subjects” variable “time” with five levels (0–30, 31–60, 61–90, 91–120, and 121–150). All post hoc comparisons were performed with the Bonferroni multiple comparisons test (corrected “alpha” 0.05/40). Calculations were made using the SPSS statistical package 17.0. A *P* value of less than 0.05 was considered statistically significant.

**Experiment 2**

Effects of RHO on the acquisition, expression, and reinstatement of cocaine-induced CPP.

### 2.3. Procedure and Experimental Design

The CPP procedure, unbiased in terms of initial spontaneous preference, was performed as described previously [18]. In short, in the first phase (preconditioning/pre-C), mice were allowed access to both compartments of the apparatus for 15 min (900 s) per day over 2 days. On day 3, the time spent in each compartment during a 900 s-period was recorded. Animals showed strong unconditioned aversion (less than 27% of the session time, i.e., 250 s) or preference (more than 73%, i.e., 650 s) for any compartment and were therefore discarded from the rest of the experimental procedure. In each group, half the animals received the drug or physiological saline in one compartment, and the other half received it in the other. After assigning animals to the compartments, an analysis of variance (ANOVA) revealed no significant differences between the time spent in the drug-paired and vehicle-paired compartments during the preconditioning phase. This is an important step in the experimental procedure that avoids any preference bias prior to conditioning. In a second phase (conditioning or acquisition) of 4 days, animals received an injection of physiological saline before being confined to the saline-paired compartment for 30 minutes, and after an interval of 4 h received an injection of 25 mg/kg of cocaine immediately before being confined to the drug-paired compartment for 30 minutes. In this phase, RHO or the vehicle was administered 60 min prior to initiation of conditioning (i.e., 60 min before the cocaine injection in the corresponding groups). Confinement was imposed in both cases by closing the guillotine door that separated the two compartments. During the third phase (postconditioning/post-C), which took place on day 8, the guillotine door separating the two compartments was removed and the time spent by the untreated mice in each compartment during a 900 s observation period was recorded. The difference in seconds between the time spent in the drug-paired compartment in the post-C test and that spent in the pre-C phase is a measure of the degree of reward induced by the drug. 

In order to evaluate the role of RHO in the acquisition of cocaine-induced CPP, eight groups of mice (*n* = 11–13 per group) were assessed. The control group received IG vehicle and one IP injection of saline (Veh + Sal), three groups received RHO 15, 20 or 25 mg/kg plus saline (RHO15, RHO 20, and RHO 25), one group received vehicle plus 25 mg/kg of cocaine (Veh + Coc), and the last three groups received RHO 15, 20, or 25 mg/kg plus 25 mg/kg of cocaine (RHO15 + Coc, RHO20 + Coc, and RHO25 + Coc). The vehicle or RHO was administered IG 60 min before cocaine or saline, which was administered IP immediately prior to four conditioning sessions. The interval of 60 min between RHO and cocaine was based on experience gained in previous studies [13], as was the absence of any interval between cocaine injection and exposure to the drug-paired compartment [18].

In order to evaluate the role of RHO in the expression of cocaine-induced CPP, four groups of mice (*n* = 9-10 per group) were conditioned with 25 mg/kg of cocaine and received the vehicle (Veh) or RHO 15, 20, or 25 mg/kg (RHO15, RHO20, and RHO25) IG 60 minutes before post-conditioning test in the colony room. 

To evaluate the effects of RHO on reinstatement of the CPP induced by re-exposure to cocaine or social defeat, we followed the procedures described previously ([18, 31], resp.). Six groups of mice (*n* = 10–13) were conditioned with 25 mg/kg of cocaine and after the post-C test, animals underwent an extinction session every 72 hours which consisted of placing the animals in the apparatus (without the guillotine doors separating the compartments) for 15 min. No drugs were administered during these sessions. The criterion for considering the preference extinguished was lack of statistical significance (according to the Student's *t*-test) between the time spent by the animals of a given group in the drug-paired compartment in the extinction session and in the pre-C session. This measure was repeated 24 h later in order to confirm the extinction. After 24 hours of confirmation to extinction, the reinstating effects of cocaine or social stress (alone or with RHO) were evaluated. Reinstatement tests were the same as those for post-C (free ambulation for 15 min), except that the animals were tested after administrating 12.5 mg/kg of cocaine or inflicting social defeat. Three groups received 12.5 mg/kg of cocaine in the colony room (a neutral place not previously associated with cocaine) 15 min before the reinstatement tests. The first group received cocaine plus vehicle (Coc), and the other two groups received cocaine plus 15 or 20 mg/kg of RHO (Coc + RHO15, Coc + RHO20), respectively (vehicle or RHO was given IG 60 min before the reinstatement test, i.e., 45 min prior to the cocaine IP injection). The other three groups of mice, exposed to social defeat (SD), received vehicle, 15, or 20 mg/kg of RHO (SD, SD + RHO15 and SD + RHO20) IG 60 min before the reinstatement test, performed immediately after SD. The social stress was performed following the procedure described by Ribeiro Do Couto et al. [31]. It took place in a different room and consisted of a 10-min agonistic encounter (and 1 min of exploration) in a neutral transparent plastic cage (23 × 13.5 × 13 cm) with a defeat result for the experimental mouse. Each experimental mouse was confronted with an aggressive opponent (of equal age and body weight) with previous fighting experience and had been shown to have a high level of aggression in previous screening. This procedure can be considered a type of social stress (see [31, 32]). Experimental mice presented avoidance/flee and defensive/submissive behaviours after suffering aggression (threat and attack) from an opponent. The criterion used to define an animal as defeated was the adopting of a specific posture of defeat, characterized by an upright submissive position, limp forepaws, upwardly angled head, and retracted ears [32, 33]. Some animals excluded from the CPP procedure (*n* = 24) were used as aggressive opponents. They were housed individually, in isolation, in plastic cages (23 × 13.5 × 13 cm) for a month before experiments to induce heightened aggression [32]. All defeated mice experienced similar levels of aggression as the opponent displayed attack behaviour as soon as it saw the experimental mouse (latency < 30 s).

### 2.4. Statistical Analysis

Data of the acquisition (the time spent in the drug-paired compartment) were analysed with a mixed ANOVA, with “treatment” as a “between-subjects” variable with eight levels (Veh + Sal, RHO15, RHO20, RHO25, RHO15 + Coc, RHO20 + Coc, RHO25 + Coc) and “days” as a “within-subjects” variable with two levels (pre-C and post-C). Data of the expression were analysed with the same ANOVA, but “treatment” variable has four levels (Veh, RHO15, RHO20, RHO25). Data of the reinstatement were analysed with the same ANOVA but, “treatment” has six levels (Coc, Coc + RHO15, Coc + RHO20, SD, SD + RHO15 and SD + RHO20) and “days” four levels (pre-C, post-C, extinction and reinstatement). 

All post hoc comparisons were performed with the Bonferroni multiple comparisons test (corrected “alpha” of 0.05/16 for acquisition data, 0.05/6 for expression data, and 0.05/24 for reinstatement data). Calculations were performed using the SPSS statistical package 17.0. A *P* value of less than 0.05 was considered statistically significant.

## 3. Results

**Experiment 1**

**Figure 1:**
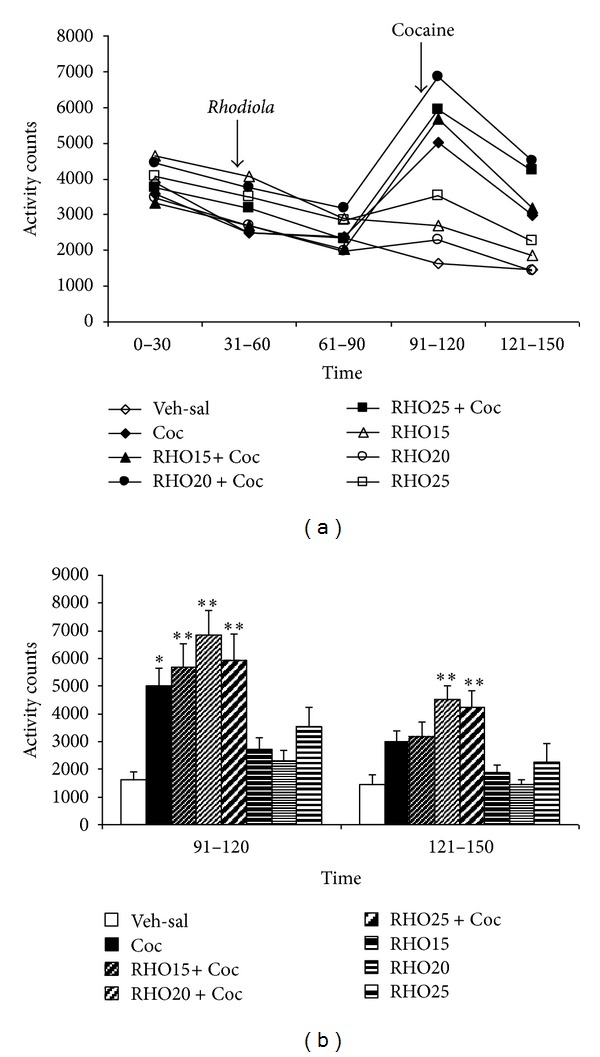
Effects of RHO on cocaine-induced hyperactivity. Mice (*n* = 8 per group) were placed in the actimeter for a 30-min adaptation period (0–30 min). Afterwards, two groups received IG vehicle (Veh-sal and Coc), two groups received IG RHO 15 mg/kg (RHO15 + Coc, RHO15), two groups RHO 20 mg/kg (RHO20 + Coc, RHO20), and two groups RHO 25 mg/kg (RHO25 + Coc, RHO25), and their motor activity was registered over another hour (31–60, 61–90 min). Finally, the first two groups received a IP injection of saline (Veh-Sal) or cocaine 25 mg/kg (Coc), and the groups treated with RHO received an IP injection of cocaine 25 mg/kg (RHO15 + Coc, RHO20 + Coc, and RHO25 + Coc) or saline (RHO 15, RHO 20, and RHO 25), and their motor activity was registered over a further hour (91–120, 121–150 min). (a) represents the motor activity of all groups over the complete time of testing (0–150 min) and shows the time of RHO and cocaine administration in the corresponding groups. (b) represents the data for the last hour of the test (after cocaine administration to the corresponding groups). Values are means ± SEM. *P < 0.05; **P < 0.01; significant difference with respect to the values of the control (Veh-sal) group at the same time test.

The results regarding the effect of RHO on cocaine-induced hyperactivity are represented in Figure 1. The ANOVA showed a significant effect of the variable time (*F*(4,224) = 43.358; *P* < 0.001), treatment (*F*(7,56) = 3.674; *P* < 0.002), and the interaction time × treatment (*F*(28,224) = 8.145; *P* < 0.001). The Bonferroni post hoc comparison showed that cocaine increased motor activity in comparison with the vehicle during the 30 min after its administration (*P* < 0.05). The groups treated with cocaine plus RHO also showed an increase in activity with respect to controls (*P* < 0.001), and the groups RHO15 + Coc and RHO20 + Coc presented more activity than the groups RHO15 and RHO20, respectively (*P* < 0.05 and *P* < 0.001). Between 31 and 60 min after cocaine administration, only the groups treated with RHO20 + Coc and RHO25 + Coc showed an increase in activity in comparison to controls (*P* < 0.001), and the group RHO20 + Coc also displayed more activity than RHO20 group (*P* < 0.001).

**Experiment 2**

**Figure 2:**
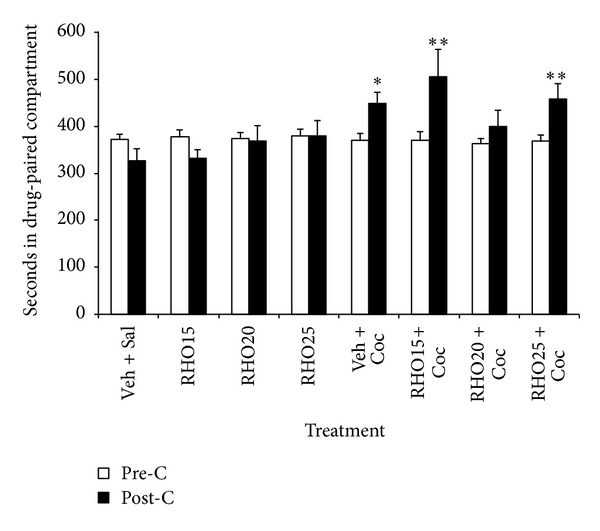
Effects of RHO on the acquisition of cocaine-induced CPP. Mice (*n* = 11–13 per group) were treated with a vehicle IG, RHO (15, 20 or 25 mg/kg, IG), cocaine (Coc 25 mg/kg, IP), or RHO 15, 20 or 25 plus Coc. RHO was administered 60 min before each saline or cocaine injection. Immediately after this second injection mice were confined to the drug-paired compartment for the conditioning phase. Bars represent the time in seconds spent in the drug-paired compartment during preconditioning (white) and postconditioning (black). Values are means ± SEM. *P < 0.05; **P < 0.01; significant difference in the time spent in preconditioning versus postconditioning sessions.

The results regarding the effect of RHO on acquisition of cocaine-induced CPP are represented in Figure 2. The ANOVA revealed a significant effect of the variable days (*F*(1,88) = 7.240; *P* < 0.01) and the interaction treatment × days (*F*(7,88) = 4.210; *P* < 0.001). Bonferroni post hoc comparisons revealed that the groups Veh + Coc, RHO15 + Coc, and RHO25 + Coc spent more time in the drug-paired compartment in post-C than in pre-C (*P* < 0.05, *P* < 0.001 and *P* < 0.01, resp.). Thus, at the doses used, RHO did not exert motivational effects, and only that of 20 mg/kg was capable of impairing the acquisition of cocaine-induced CPP.

**Figure 3:**
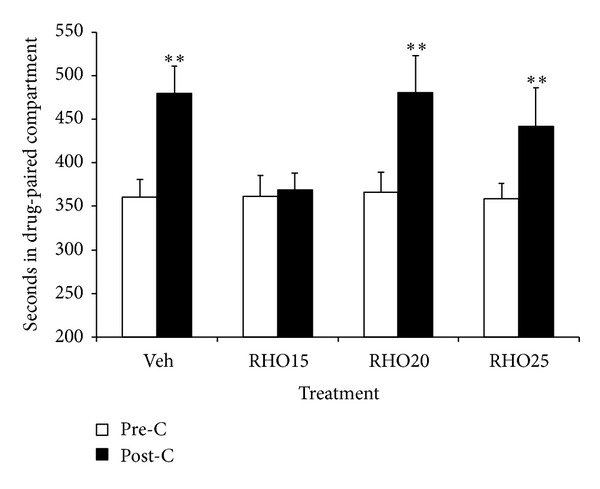
Effects of RHO on the expression of cocaine-induced CPP. Mice (*n* = 9-10 per group) were conditioned with Cocaine (Coc 25 mg/kg, IP) and received IG Veh or RHO (15, 20 or 25 mg/kg) 60 min before the post-conditioning test. Bars represent the time in seconds spent in the drug-paired compartment during preconditioning (white) and post-conditioning (black). Values are means ± SEM. **P < 0.01; significant difference in the time spent in preconditioning versus post-conditioning sessions.

The effects of RHO on the expression of cocaine-induced CPP are represented in Figure 3. The ANOVA revealed a significant effect of the variable days (*F*(1,35) = 33.264; *P* < 0.001) and the interaction treatment × days (*F*(3,35) = 3.378; *P* < 0.05). Bonferroni post hoc comparisons revealed that the groups receiving vehicle or RHO 20 or 25 mg/kg spent more time in the drug-paired compartment in post-C than in pre-C (*P* < 0.001, *P* < 0.001, and *P* < 0.005, resp.). In this way, only the dose of 15 mg/kg of RHO blocked the expression of cocaine-induced CPP.

**Figure 4:**
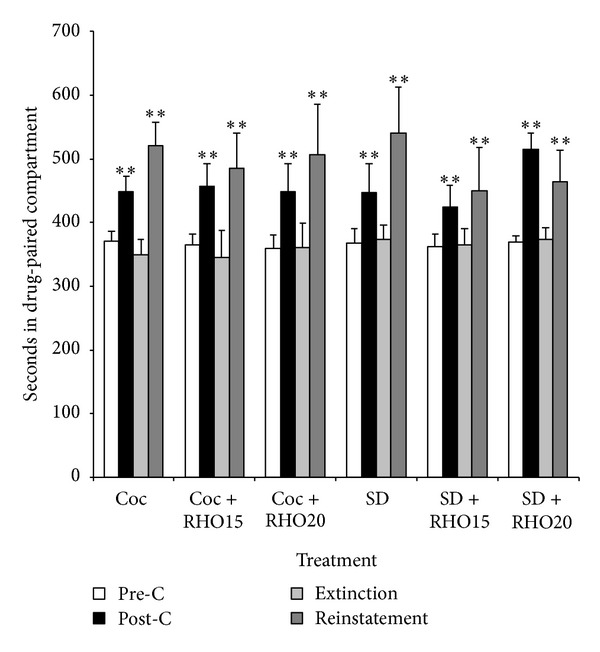
Effects of RHO on the priming- and stress-induced reinstatement of cocaine-induced CPP. Six groups of mice (*n* = 10–13 for group) were conditioned with cocaine (Coc 25 mg/kg, IP), underwent daily extinction sessions until the CPP was extinguished, and received the following treatments before the reinstatement test: vehicle, RHO 15, or 20 mg/kg 45 min before 12.5 mg/kg IP cocaine priming (Coc, Coc + RHO15, Coc + RHO20); vehicle, RHO 15, or 20 mg/kg 45 min before social defeat exposure (SD, SD + RHO15, SD + RHO20). Bars represent the time in seconds spent in the drug-paired compartment before conditioning sessions in the pre-C test (white bars), after conditioning sessions in the post-C test (black bars), in the last extinction session (light gray bars), and in the reinstatement test (dark gray bars). **P < 0.001, significant difference in the time spent in the drug-paired compartment in preconditioning versus post-conditioning or reinstatement test.

The effects of RHO on the priming- and social defeat-induced reinstatement of cocaine CPP are represented in Figure 4. The ANOVA revealed a significant effect of the variable days (*F*(3,59) = 26.101; *P* < 0.001). Bonferroni post hoc comparisons revealed that more time was spent in the drug-paired compartment during post-C and the reinstatement test than during pre-C or extinction. The variables treatment and interaction were not significant. These results show that RHO does not block the reinstatement of cocaine-CPP induced by priming or stress.

## 4. Discussion

The results obtained in this study demonstrate that RHO is capable of decreasing the rewarding effects of cocaine, since both acquisition and expression of a cocaine-induced CPP were impaired by RHO, though only with specific doses of this compound. However, cocaine-induced hyperactivity and the reinstatement of cocaine CPP induced by priming or stress were not blocked by RHO. These results provide evidence that RHO does not block all the behavioural effects of cocaine and that the impairing effects of RHO on the rewarding actions of cocaine are in function of the dose used and the conditioning process studied.

First, we have seen that RHO does not block the hyperactivity induced by cocaine and even seems to increase the duration of this effect, since animals treated with cocaine plus the intermediate and high doses of RHO showed hyperactivity when the stimulant motor effects of cocaine were insignificant (between 30 and 60 minutes after administration). Many of the behavioural effects of cocaine, including its locomotor-activating properties, have been attributed to the ability of this drug to block DA transporters and enhance DA activity [34]. Our results suggest that RHO increases DA levels slightly. This effect was not sufficient to significantly modify motor activity in the animals treated with RHO alone but together with the stimulatory effects of cocaine induced a marked hyperactivity. 

The CPP paradigm has been used to evaluate the rewarding action of drugs of abuse, including morphine and cocaine [14–16]. We observed that cocaine induced rewarding effects, in accordance with previous studies in our laboratory [18]. Conversely, the administration of RHO did not induce motivational effects, which is also in line with previous reports [13]. The administration of RHO impaired the acquisition and expression of cocaine-induced CPP, although these effects were observed only with specific doses of this compound (20 mg/kg blocked acquisition and 15 mg/kg blocked expression). Thus, these partial effects of RHO on cocaine-induced CPP could be related to the dose employed. The effect of *Rhodiola* on the CNS and other body systems did not vary in a consistent manner with the dose. The dose-dependent curve has a bell shape, *Rhodiola* is inactive at small doses, is active at intermediate doses, and becomes inactive again at high doses [10]. The fact that RHO did not induce aversive or motor effects by itself suggested that it prevents cocaine CPP selectively, thus undermining the rewarding effects of this drug. The possibility that RHO impairs learning and memory of cocaine conditioning is improbable, since RHO is known to exert a positive influence on the development of conditioned reflexes and learning [35, 36]. Our results are only partially in agreement with those of Mattioli et al. [13], since they demonstrated that different doses of RHO (10, 15 and 20 mg/kg) impaired the acquisition and expression of morphine CPP. Thus, RHO seems to have more effectiveness in decrease opiate than cocaine reward. The precise mechanism underlying the efficacy of RHO in blocking morphine-induced CPP is unknown, but Mattioli et al. [13] speculate that RHO could exert this effect by enhancing the functional tone of endogenous opioids and 5-HT, DA, and NA in brain areas related to reward [6, 27]. Differences in the mechanism of action of morphine and cocaine and in the behavioural effects of these drugs [30] can explain why RHO is less effective to reduce cocaine reward. Cocaine is a psychomotor stimulant that facilitates monoaminergic neurotransmission by binding to DA, 5-HT, and NA transporters (DAT, SERT, and NAT) and inhibiting the reuptake of these monoamines. Cocaine produces reward through simultaneous actions at more than one protein site [34, 37]. For example, only DAT/SERT knockout mice exhibit impairment of cocaine CPP [37] and the contribution of DA, 5-HT, and NA transporters to the rewarding, and aversive effects of cocaine seem to vary [34]. Activation of the mesolimbic dopaminergic reward circuitry has been proposed as a modality in the long-term treatment of reward deficiency syndrome and drug addiction disorders [38]. In addition to the abovementioned dose problem, the fact that RHO impairs acquisition or expression of cocaine CPP only at specific doses could be related to the effects that this compound induces monoamines (DA, 5-HT, and NA) and different kinds of monoaminergic precursors, receptors, transporters, or degradation enzymes. In such circumstances, impairment of acquisition/expression of cocaine CPP would be induced only by doses of RHO that produce an effective combination of alterations in the various monoamine proteins involved in the acquisition and/or expression of the rewarding effects of cocaine.

Although the positive reinforcing is the main factor in the acquisition of a drug habit, relapse is the overriding characteristic of addiction and the foremost challenge to the treatment of drug addiction. The CPP paradigm can be used to model relapse in rodents, and re-exposure to drug or stress after extinction can trigger reinstatement of CPP [17]. We observed that cocaine CPP is reinstated by cocaine priming and social defeat stress in accordance with previous studies [18, 26]. However, the administration of RHO did not block priming- or stress-induced reinstatement. These results contrast with those obtained by Mattioli et al. [13] who reported that RHO prevents the reinstatement of morphine CPP induced by drug re-exposure and restraint stress. Again, these divergent results could be related to the different mechanisms underlying the reinstatement of CPP induced by morphine or cocaine. It is improbable that the increase in monoaminergic activity induced by RHO blocks priming-induced reinstatement of cocaine CPP since it has been reported that administration of the DA D1 agonist SKF 81297 [39] and the DA enhancer modafinil [40] induces the reinstatement of cocaine CPP. On the other hand, the lack of effects of RHO on stress-induced reinstatement of cocaine CPP is surprising, since this compound exerts antistress effects and interacts with the hypothalamus-pituitary-adrenal (HPA) system, reducing cortisol levels [7–9]. However, it has been reported that corticosterone plays at most a permissive role in the facilitating effects of social stress on cocaine self-administration in the rat [41] and that stress-induced reinstatement of cocaine self-administration appears to be independent of corticosterone [42]. Alternatively, it is possible that a single administration of an acute dose of RHO prior to reinstatement tests is insufficient to decrease to attenuate the reinstatement of cocaine CPP. 

The doses employed in the present study are in the range of those administered to humans for therapeutic purposes, although clinical studies have tended to use higher doses [43, 44]. According to the calculating method described by Reagan-Shaw et al. [45], in a human weighing 60 kg, 15 mg/kg of RHO corresponds with 72.9 mg, 20 mg/kg corresponds with 97.2 mg, and 25 mg/kg corresponds with 120 mg. Extracts of RHO have already been used in humans without severe adverse consequences, which endorses the safety of the preparations in question [5, 9, 35, 43, 44, 46–48]. Only a few mild adverse events have been reported, including headache or hypersalivation [43]. Moreover, a lack of interaction with other drugs (warfarin and theophylline) has been observed [49]. However, it remains to be determined whether or not RHO interacts with cocaine or other drugs in clinical settings.

Presently, there are no FDA-approved therapies or medications for treating cocaine addiction [2, 3] that can be used as a positive control in the evaluation of RHO's effects. However, there are recent reports of the positive effects of derivatives of genera Stephania and Corydalis in the treatment of cocaine addiction in animal models. Levo-tetrahydropalmatine has been shown to attenuate cocaine self-administration [50] and reinstatement of extinguished cocaine seeking by cocaine, stress, or drug-associated cues in rats [50, 51]. In light of this evidence, levo-tetrahydropalmatine has been suggested as a potential new medication for the treatment of cocaine addiction [52].

The main limitation of our work lies in the fact that RHO was administered over a short period of a few days (in four acquisition sessions) or acutely (in expression and reinstatement tests). It is likely that more pronounced effects of RHO would be observed with a longer period of administration (e.g., during the extinction period). Another limitation of our approach is the use of a single animal model to evaluate the rewarding effects of cocaine. The CPP paradigm used in the present study measures the conditioned rewarding effects of the drug while the drug self-administration paradigm measures the primary hedonic properties of the drug [17]. Future research should evaluate the efficacy of RHO in reducing the acquisition of cocaine self-administration and reinstatement of drug seeking after exposure to cocaine priming, drug-conditioned cues, or stress. 

In conclusion, we can report that RHO impairs the rewarding effects of cocaine, although its effects on acquisition and expression of CPP depend on the dose used. The fact that RHO does not affect priming- or drug-induced reinstatement of cocaine CPP limits its possible usefulness as a natural treatment for cocaine dependence. Nevertheless, though the discovery of an effective therapy that addresses all aspects of cocaine addiction continues to elude researchers, the anti-craving effect of RHO shows potential as a component of combined therapy.
